# An Internet-Based Physical Activity Intervention to Improve Quality of Life of Inactive Older Adults: A Randomized Controlled Trial

**DOI:** 10.2196/jmir.4335

**Published:** 2016-04-27

**Authors:** Karen Broekhuizen, Jelle de Gelder, Carolien A Wijsman, Liselotte W Wijsman, Rudi GJ Westendorp, Evert Verhagen, Pieternella E Slagboom, Anton J de Craen, Willem van Mechelen, Diana van Heemst, Frans van der Ouderaa, Simon P Mooijaart

**Affiliations:** ^1^ Leiden University Medical Center Department of Gerontology and Geriatrics Leiden Netherlands; ^2^ Institute for Evidence-Based Medicine in Old Age Leiden Netherlands; ^3^ Leyden Academy on Vitality and Ageing Leiden Netherlands; ^4^ ExtraMuraal Gezondheids Onderzoek (EMGO+) Institute for Health and Care Research Department of Public and Occupational Health Vrije Universiteit (VU) University Medical Center Amsterdam Netherlands; ^5^ Department of Medical Statistics, Section of Molecular Epidemiology Leiden University Medical Center Leiden Netherlands

**Keywords:** Internet, physical activity, quality of life

## Abstract

**Background:**

Increasing physical activity is a viable strategy for improving both the health and quality of life of older adults.

**Objective:**

The aim of this study was to assess if an Internet-based intervention aimed to increase physical activity was effective in improving quality of life of inactive older adults. In addition, we analyzed the effect of the intervention on quality of life among those participants who successfully reached their individually targeted increase in daily physical activity as indicated by the intervention program, as well as the dose-response effect of increasing physical activity on quality of life.

**Methods:**

The intervention was tested in a randomized controlled trial and was comprised of an Internet program—DirectLife (Philips)—aimed at increasing physical activity using monitoring and feedback by accelerometry and feedback by digital coaching (n=119). The control group received no intervention (n=116). Participants were inactive 60-70-year-olds and were recruited from the general population. Quality of life and physical activity were measured at baseline and after 3 months using the Research ANd Development 36-item health survey (RAND-36) and wrist-worn triaxial accelerometer, respectively.

**Results:**

After 3 months, a significant improvement in quality of life was seen in the intervention group compared to the control group for RAND-36 subscales on emotional and mental health (2.52 vs -0.72, respectively; *P*=.03) and health change (8.99 vs 2.03, respectively; *P*=.01). A total of 50 of the 119 participants (42.0%) in the intervention group successfully reached their physical activity target and showed a significant improvement in quality of life compared to the control group for subscales on emotional and mental health (4.31 vs -0.72, respectively; *P*=.009) and health change (11.06 vs 2.03, respectively; *P*=.004). The dose-response analysis showed that there was a significant association between increase in minutes spent in moderate-to-vigorous physical activity (MVPA) and increase in quality of life.

**Conclusions:**

Our study shows that an Internet-based physical activity program was effective in improving quality of life in 60-70-year-olds after 3 months, particularly in participants that reached their individually targeted increase in daily physical activity.

**Trial Registration:**

Nederlands Trial Register: NTR 3045; http://www.trialregister.nl/trialreg/admin/rctview.asp?TC=3045 (Archived by WebCite at http://www.webcitation.org/6fobg2sjJ)

## Introduction

Increasing physical activity is a viable strategy for improving both health and quality of life in inactive older adults, who are a growing public health concern [1]. It is estimated that the proportion of adults aged 65 years and over will account for about 11% (939 million) of the total global population by 2030 [2]. Increased life expectancy is associated with an increase in multiple chronic conditions, translating into functional disability, need for assistance, reduced mobility, depression, isolation, and loneliness [3]. These outcomes are related to functioning and well-being and fall under the umbrella term *quality of life* [4]. Earlier efforts of health promotion have primarily focused on lower mortality rates or reduced disease risk. In the past decade, there is increasing concern that quality of life deserves attention as well [5].

Previously, we investigated the effects of a 3-month Internet-assisted intervention directed at increasing daily physical activity on objectively measured physical activity and metabolic health in 60-70-year-old inactive individuals. The intervention was tested in a randomized controlled trial and comprised of an Internet program—DirectLife (Philips)—aimed at increasing physical activity using monitoring and feedback by accelerometry and feedback by digital coaching. Results showed that the intervention was effective in increasing physical activity and in improving metabolic health [6,7].

In this study, our aim was threefold. First, we aimed to assess if the intervention was also effective in improving quality of life. Second, we analyzed the effect on quality of life among those participants who successfully reached their individually targeted increase in daily physical activity as indicated by the DirectLife program. Finally, we performed a dose-response analysis of increasing physical activity on quality of life among all participants.

## Methods

### Overview

Analyses were performed with data obtained from a previously reported randomized controlled trial—Nederlands Trial Register: NTR 3045—on the effects of a 3-month Internet-assisted intervention directed at increasing daily physical activity on objectively measured physical activity and metabolic health in 60-70-year-old inactive individuals. The CONSORT-EHEALTH Checklist for this trial is included as Multimedia Appendix 1 [8]. Details on study design and intervention content have been published elsewhere [6]. In short, the study recruited inactive participants aged 60-70 years from the region of Leiden, the Netherlands. The presence of an inactive lifestyle was assessed before randomization by a self-reported physical activity questionnaire: the General Practice Physical Activity Questionnaire (GPPAQ) [9]. This yielded four categories of physical activity: inactive, moderately inactive, moderately active, and active. We defined inactive as having less than 3 hours per week of exercise and cycling combined, corresponding to the inactive, moderately inactive, or moderately active category. Participants in the active category of the GPPAQ did not meet inclusion criteria for our definition of an inactive lifestyle. Participants were considered eligible if they (1) had no history of diabetes or did not use glucose-lowering medication, (2) had no disability impeding increase in physical activity, and (3) possessed and used a personal computer with Internet connection. At baseline, participants were randomly assigned to the intervention group or to a waiting list control group by the study physician or research nurse. Participants were randomized, via computerized program, into intervention versus waiting list control groups at a ratio of 1:1, with a block size of 12; stratification was performed by sex. Concealment of treatment allocation was ensured by randomizing at the end of the first study visit, after all baseline measurements and instructions at the study center were completed. Written informed consent was obtained from all participants. The study was approved by the Medical Ethical Committee of Leiden University Medical Center, the Netherlands.

### Intervention

Participants in the intervention group received a commercially available Internet-based physical activity program—DirectLife (Philips, Consumer Lifestyle, Amsterdam, the Netherlands)—directed at increasing daily physical activity. The DirectLife program comprised components that are based on the stages of change and the I-Change model. Briefly, this model assumes that behavioral change is the result of individual awareness of one's behavior, motivation to change behavior and action, and taking into account the individual’s current activity level; it subsequently provides a personal goal [6,10,11]. DirectLife consists of three elements: (1) an accelerometer-based activity monitor, (2) a personal website, and (3) a personal e-coach, who provides regular updates of the individual’s physical activity status by email and gives advice on how to increase physical activity. By means of these elements, the program aims to increase awareness about one’s own physical activity behavior, to give feedback on recent actual physical activity, and to provide support to make sustainable changes in physical activity behavior. The activity monitor of DirectLife is based on the Tracmor triaxial accelerometer, and has been validated against double-labeled water for the estimation of total daily life energy expenditure [12]. The DirectLife monitor is the consumer version of the Tracmor accelerometer. Participants of the program were instructed to wear the activity monitor continuously throughout the day to measure daily physical activity. Data were uploaded through a secure Internet connection to the database of the commercial provider. After an initial 8-day *assessment period* starting 1 week after the study visit, in which the current level of daily activity was measured, a target was set to increase the level of daily activity during a 12-week Internet-based interactive coaching program. Personalized targets were set by the DirectLife program and were defined as the absolute increase in physical activity compared to the individual’s baseline assessment data. For the whole group, this corresponded to a mean increase of approximately 10% in daily physical activity at week 12, increasing at a linear rate per week. All participants were given the option to decrease the personalized goal, within limits (ie, minimum of 5% increase in physical activity versus 10%), or to increase their personalized end goal, dependent on physical activity level of the last week.

Participants were given a target for daily activity, which increased weekly, and data from the accelerometer were used for regular feedback. Coaching included general recommendations on physical activities and coaches were available for further questions and advice by email correspondence. The control group was placed on a 3-month waiting list after which they received access to the intervention program at the end of the study. During the trial, no specific instructions regarding daily physical activity were given to the control group.

### Measurements

Enrollment and follow-up took place from November 2011 to August 2012.

#### Baseline Questionnaire

In preparation of the first visit to the study center, all participants completed an Internet-delivered questionnaire on education, smoking status, and medical history, including medication use. Education was categorized as low (primary education and lower vocational education), intermediate (secondary education and intermediate vocational education), or high (high vocational education and university).

#### Quality of Life

Health-related quality of life was assessed at baseline and at 3-month follow-up, with the use of the self-administered standard Dutch paper version of the Research ANd Development (RAND) 36-item health survey (RAND-36) [13]. The RAND-36 questionnaire entails eight domains of health-related quality of life pertaining to both physical and mental health. The domains of physical functioning (10 items), limitations on usual role-related activities due to physical health problems (four items), pain (two items), and general health perception (five items) comprise the physical component; the domains of vitality (four items), social functioning (two items), limitations on usual role-related activities due to emotional or mental problems (three items), and emotional or mental problems (three items) comprise the mental component. In addition to the eight subscales, participants were asked to compare their current general health with their general health one year earlier. Scores on the subscales range from 0 to 100, with higher scores indicating better health or functioning. The total RAND-36 score is the sum of the scores on the subscales and ranges from 0 to 800, where 0 is the poorest quality of life and 800 the best imaginable. The RAND-36 is a reliable and valid measure for determining health-related quality of life in the elderly [13].

#### Physical Activity

The primary outcome was the individual's relative change in activity counts after the intervention compared to baseline, measured at the right wrist by the activity monitor. At baseline and 3-month follow-up, daily physical activity was measured during 7 days following the visit at the study center, using a wrist-worn triaxial accelerometer—GeneActiv (Kimbolton, Cambridgeshire, UK). To assess the primary outcome, we used accelerometers other than the one included in the intervention program to avoid interpretation of the intervention as an outcome. As a derivative outcome, we calculated from the wrist accelerometer the minutes per day spent in moderate-to-vigorous physical activity (MVPA), which has been validated against indirect calorimetry [14]. We chose not to report physical activity in counts, but in the amount of minutes spent in MVPA. For the elderly, general recommendations entail at least half an hour of MVPA at least five days a week. While the recommendations are formulated in terms of minutes spent in MVPA, the outcome of our study is better interpretable in terms of guideline adherence. In addition, minutes spent in MVPA as a derivative outcome for activity counts has been validated against indirect calorimetry. A detailed description of processing the collected accelerometer data into activity counts and average number of minutes, daily, spent in MVPA is described elsewhere [6]. Outcome assessment was done by an independent researcher who was blind to study arm allocation.

#### Successful Use of DirectLife Program

From the intervention group, a subgroup was created including participants who successfully reached their individually targeted increase in daily physical activity as indicated by the intervention program. An average level of physical activity per week was calculated from the last 3 weeks of the program and was compared with the personalized target of the corresponding week. Because a substantial number of participants reached the targeted personalized goals at the end of the 12-week program, but with some variation in the last 3 weeks, we labeled participants as being successful if they reached their target in at least 2 of the 3 last weeks of the program [7].

### Statistical Analyses

Normally distributed data are shown as means with standard deviation, skewed data as medians with interquartile range (IQR). Between-group differences in quality of life after 3 months were analyzed by the intention-to-treat principle with an independent samples *t* test. For relative change in MVPA, the Mann-Whitney nonparametric test was performed. In a secondary analysis, we included only those participants in the intervention group who successfully reached the individual, personalized end goal to increase average physical activity that was set as part of the intervention program. To investigate if an increase in physical activity was associated with an improvement in quality of life, linear regression models were used, adjusted for age, sex, and body mass index (BMI) [15]. For this purpose, physical activity was divided into tertiles based on the change in minutes spent in MVPA. All analyses were performed with SPSS version 20.0 (IBM Corp, Armonk, NY, USA). Statistical significance was accepted at *P*<.05.

## Results

**Figure 1:**
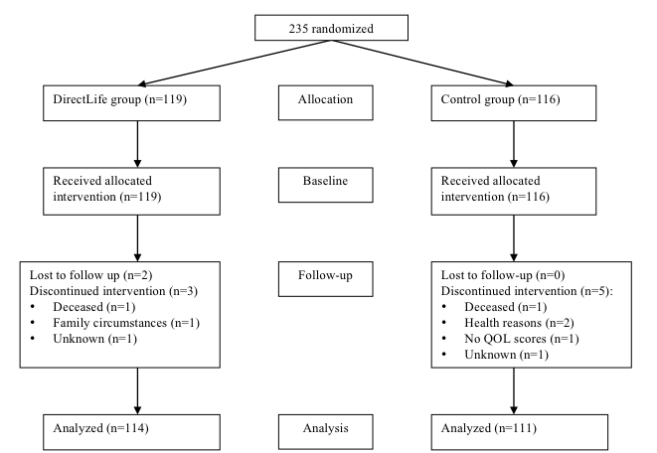
Flowchart of participants. QOL: quality of life.

Figure 1 shows the flowchart of included participants. A total of 235 participants were randomized—119/235 (50.6%) intervention, 116/235 (49.4%) control—and 96.2% (226/235) completed the trial—114/226 (50.4%) intervention, 111/226 (49.1%) control. Wijsman and colleagues already reported the main intervention effects [6]. In short, significant changes in favor of the intervention group were found for minutes of MVPA, weight loss, fat percentage, and glycated hemoglobin (HbA1c) [6]. In addition, in a dose-response analysis, Vroege and colleagues showed that there was a significant association between an increase in minutes spent in MVPA and body weight loss, reduction of BMI, waist circumference reduction, increase of high-density lipoprotein (HDL) cholesterol, and lowering of low-density lipoprotein (LDL)/HDL ratio [7].

Table 1 shows the baseline characteristics of the 235 study participants. The intervention and control groups were similar for all characteristics. In both groups, most participants were male, with a mean age of approximately 65 years old. The study population was overweight, with a mean BMI of 28.9 kg/m^2^ (SD 4.7) and 29.1 kg/m^2^ (SD 4.7) in the intervention and control groups, respectively.

Table 2 describes the change in quality of life for both the intervention and control groups. After 3 months, a significant improvement in quality of life was seen in the intervention group compared to the control group for subscales on emotional and mental health (2.52 vs -0.72, respectively; *P*=.03, 95% CI 0.39-6.09) and health change (8.99 vs 2.03, respectively; *P*=.01, 95% CI 1.60-12.32). No significant between-group differences were found for all other subscales, nor for the total RAND-36 score. From the results of the main analyses of Wijsman and colleagues, we know that accelerometer data were available for 107 intervention and 109 control participants, and that after 3 months, there was a mean increase of 11.1 minutes (SE 2.1) of MVPA per day in the intervention group, compared to a mean decrease of 0.1 minutes (SE 1.5) in the control group (*P*=.001) [6].

Further analysis included only those participants in the intervention group who were successful in reaching their personalized target after finishing the 3-month intervention program. This was the case for 50 (42.0%) of the 119 participants in the intervention group. Similar to the results from our primary analysis, a significant improvement in quality of life was seen in the successful intervention group compared to the control group for subscales on emotional and mental health (4.31 vs -0.72, respectively; *P*=.009, 95% CI 1.26-8.79) and health change (11.06 vs 2.03, respectively; *P*=.004, 95% CI 2.93-15.13). Overall, improvements in quality of life were larger in the successful intervention group compared to the overall intervention group for all subscales, as well as for the total RAND-36 score (see Table 2).

Table 3 describes the relationship between increase in physical activity and improvement in quality of life. Increase in physical activity in the entire sample was divided into tertiles based on the change in minutes spent in MVPA (see Table 3). Because of technical errors, data on activity counts were available for only 211 of the 235 participants (89.8%). With an increase of MVPA, the total RAND-36 score improved significantly (*P*_trend_=.001, 95% CI 0.02-0.09), as well as quality of life regarding the subscales' usual role-related activities due to emotional health problems (*P*_trend_ =.03, 95% CI 0.01-0.20), emotional or mental health (*P*_trend_=.005, 95% CI 0.10-0.58), pain (*P*_trend_=.008, 95% CI 0.06-0.38), vitality (*P*_trend_=.004, 95% CI 0.11-0.54), and general health perception (*P*_trend_=.04, 95% CI 0.01-0.41). Other subscales were not associated with an increase in MVPA.

**Table 1:** Baseline characteristics of 235 study participants.

Characteristics			Intervention group (n=119)	Control group (n=116)
**Demographics**				
	Sex (female), n (%)		47 (39.5)	49 (42.2)
	Age (years), mean (SD)		64.7 (3.0)	64.9 (2.8)
	**Degree of self-reported activity, n (%)**			
		Moderately active	41 (34.5)	48 (41.4)
		Moderately inactive	36 (30.3)	34 (29.3)
		Inactive	42 (35.3)	34 (29.3)
	**Level of education, n (%)**			
		Low	7 (5.9)	2 (1.7)
		Intermediate	45 (38.1)	46 (40.0)
		High	66 (55.9)	67 (58.3)
**Clinical parameters, mean (SD)**				
	Height (cm)		173.6 (9.9)	172.1 (9.3)
	Weight (kg)		87.4 (15.8)	86.3 (15.8)
	BMI^a^ (kg/m^2^)		28.9 (4.7)	29.1 (4.7)
**Physical activity per day, mean (SD)**				
	Minutes spent in moderate-to-vigorous activity, median (IQR^b^)		16.8 (18.6)	14.4 (23.8)
**Quality of life,** ^c^ **mean (SD)**				
	Physical functioning		83.40 (14.98)	84.61 (15.05)
	Social functioning		88.03 (17.63)	86.42 (18.76)
	Role limitations (physical problem)		81.30 (32.09)	82.97 (29.41)
	Role limitations (emotional problem)		85.99 (28.95)	86.21 (28.51)
	Emotional or mental health		77.24 (15.52)	77.31 (15.58)
	Vitality		67.48 (17.07)	67.03 (17.69)
	Pain		80.04 (20.20)	84.29 (17.03)
	General health perception		68.15 (16.34)	67.72 (14.94)
	Health change		53.57 (20.40)	51.51 (13.31)
	Total RAND-36^d,e^ score		630.86 (120.12)	639.68 (118.64)

^a^BMI: body mass index.

^b^IQR: interquartile range.

^c^Scores on the subscales of quality of life range from 0 to 100, with higher scores indicating better health or functioning.

^d^RAND-36: Research ANd Development 36-item health survey.

^e^The total RAND-36 score is the sum of the scores on the subscales and ranges from 0 to 800.

**Table 2:** Changes in quality of life in the control, intervention, and successful intervention group.

Quality-of-life subscales^a^	All (n=225)	Control group (n=111)	Intervention group (n=114)		Successful intervention group (n=50)	
	Mean (SE)	Mean (SE)	Mean (SE)	*P* (95% CI) (between groups^b,c^)	Mean (SE)	*P* (95% CI) (between groups^b,d^)
Δ^e^ Physical functioning	1.40 (0.77)	0.95 (1.01)	1.84 (1.16)	.56 (-2.14 to 3.93)	3.37 (1.60)	.19 (-1.21 to 6.06)
Δ Social functioning	-0.44 (1.06)	-1.13 (1.62)	0.22 (1.37)	.53 (-2.82 to 5.51)	1.20 (1.95)	.39 (-3.04 to 7.69)
Δ Role limitations (physical problem)	0.11 (1.94)	2.03 (2.93)	-1.75 (2.56)	.33 (-11.44 to 3.88)	-1.92 (4.28)	.45 (-14.20 to 6.30)
Δ Role limitations (emotional problem)	-1.04 (1.97)	-2.40 (2.62)	0.29 (2.95)	.50 (-5.09 to 10.48)	0 (3.88)	.61 (-6.80 to 11.60)
Δ Emotional or mental health	0.92 (0.73)	-0.72 (1.19)	2.52 (0.83)	*.03* ^f^ (0.39 to 6.09)	4.31 (1.14)	*.009*(1.26 to 8.79)
Δ Vitality	1.84 (0.83)	0.90 (1.09)	2.76 (1.24)	.26 (-1.40 to 5.13)	4.62 (1.89)	.07 (-0.34 to 7.77)
Δ Pain	-0.12 (1.09)	-0.74 (1.50)	0.48 (1.58)	.58 (-3.07 to 5.51)	-0.47 (2.04)	.92 (-4.87 to 5.40)
Δ General health perception	0.42 (0.77)	0.22 (1.22)	-0.09 (2.29)	.86 (-3.83 to 3.21)	0.38 (3.32)	.94 (-4.22 to 4.54)
Δ Health change	5.56 (1.38)	2.03 (1.44)	8.99 (2.29)	*.01*(1.60 to 12.32)	11.06 (2.28)	*.004*(2.93 to 15.13)
Δ Total RAND-36^g,h^ score	2.75 (5.55)	-0.89 (7.57)	6.28 (8.13)	.52 (-14.75 to 29.09)	11.48 (11.96)	.37 (-14.80 to 35.54)

^a^Scores on the subscales range from 0 to 100, with higher scores indicating better health or functioning.

^b^Independent samples *t* test.

^c^Difference between control and intervention group.

^d^Difference between control and successful intervention group.

^e^Represents change in subscale score from baseline to follow-up.

^f^ *P* values in italics represent significant values.

^g^RAND-36: Research ANd Development 36-item health survey.

^h^The total RAND-36 score is the sum of the scores on the subscales and ranges from 0 to 800.

**Table 3:** Dose-response relationship of the change in minutes spent in moderate-to-vigorous physical activity with quality of life.

Characteristics		Tertiles of Δ^a^ moderate-to-vigorous physical activity (minutes per day)			
		Low (n=69)	Middle (n=72)	High (n=70)	*P* ^b^ (95% CI)	
Δ Moderate-to-vigorous physical activity (minutes per day), median (IQR^c^)		-7.80 (10.65)	2.20 (3.20)	19.40 (20.00)	N/A^d^
**Quality-of-life subscales,** ^e^ **mean (SD)**					
	Δ Physical functioning	1.64 (1.39)	0.73 (1.36)	2.07 (1.38)	.26 (-0.10 to 0.37)
	Δ Social functioning	-1.22 (1.88)	-0.84 (1.84)	-1.68 (1.86)	.46 (-0.11 to 0.24)
	Δ Role limitations (physical problem)	-4.01 (3.60)	1.15 (3.51)	3.48 (3.55)	.09 (-0.01 to 0.17)
	Δ Role limitations (emotional problem)	-3.62 (3.49)	-2.73 (3.41)	0.66 (3.45)	*.03* ^f^ (0.01 to 0.20)
	Δ Emotional or mental health	-0.73 (1.35)	0.78 (1.32)	2.26 (1.33)	*.005* (0.10 to 0.58)
	Δ Vitality	1.98 (1.49)	0.62 (1.45)	3.55 (1.47)	*.004* (0.11 to 0.54)
	Δ Pain	-2.53 (1.97)	0.47 (1.93)	3.00 (1.95)	*.008* (0.06 to 0.38)
	Δ General health perception	-2.29 (1.62)	1.07 (1.59)	1.44 (1.60)	.*04* (0.01 to 0.41)
	Δ Health change	5.14 (2.40)	2.37 (2.35)	9.29 (2.37)	.07 (-0.01 to 0.26)
	Δ Total RAND-36^g,h^ score	-10.77 (9.93)	1.24 (9.71)	14.79 (9.81)	.*001* (0.02 to 0.09)

^a^Represents change in values from baseline to follow-up.

^b^Linear regression, adjusted for sex, age, and body mass index (BMI).

^c^IQR: interquartile range.

^d^N/A: not applicable.

^e^Scores on the subscales range from 0 to 100, with higher scores indicating better health or functioning.

^f^ *P* values in italics represent significant values.

^g^RAND-36: Research ANd Development 36-item health survey.

^h^The total RAND-36 score is the sum of the scores on the subscales and ranges from 0 to 800.

## Discussion

### Principal Findings

In this randomized controlled trial assessing the effect of an Internet-based intervention on improving quality of life in inactive older adults, we found a significant improvement in quality of life in the intervention group compared to the control group for subscales on *emotional or mental health* and *health change* of the RAND-36 health survey. Improvements in quality of life were particularly observed in those participants in the intervention group who had successfully reached their personalized target with the intervention. Furthermore, our results show that more MVPA is associated with higher quality-of-life levels.

Earlier, we found that the DirectLife intervention led to significant improvements of metabolic health and showed a significant association between an increase in minutes spent in MVPA and metabolic health outcomes [6,7]. Given the observed dose-response relationship we have shown between physical activity and the majority of subscales of quality of life, we are confident that the DirectLife program has induced an improvement in quality of life through an increase in physical activity. Our results are in accordance with results from previous experimental studies that showed that an increase in physical activity had a positive effect on quality of life in older adults [16,17]. These studies performed no subgroup analyses that included successful intervention adopters only. Evidence from a series of randomized controlled trials conducted with frail older adults to test the effects of physical activity on quality of life has indicated that there is a positive effect on emotional and social functioning in particular, and that physical activity did not exacerbate perceptions of pain [18]. Our results also confirm these findings, as the intervention particularly induced an improvement of mentally related subscales of the RAND-36 and did not lead to increased perceptions of pain. It is actually noteworthy that the effects of DirectLife induced the largest increases in subscales regarding the emotional component of quality of life. One of the explanations for this might be the type of intervention. DirectLife primarily focused on personal goal setting, aiming for an increase of perceived control, self-efficacy, and mastery, which probably will induce an improvement of mental functioning in particular [1,19,20]. Secondly, our sample contained inactive older people, who will probably more directly experience improvement of mental functioning, rather than physical functioning. As the empirical evidence regarding the mechanisms underlying the association between increased physical activity and quality of life is limited, these hypotheses remain speculations and further research on this topic is warranted.

Assuming that an increase of ≥5 points in any of the RAND-36 subscales is clinically relevant [21], the clinical relevance of the significant increases in the *emotional or mental health* (2.52 points) and *health change* (8.99 points) subscales in the intervention group is moderate to high. Baseline scores on the RAND-36 were higher than scores observed in a healthy Dutch population in a similar age category, particularly for physical functioning and limitations on usual role-related activities due to physical health problems [13]. Apparently, inactive 60-70-year-olds do not feel hampered in daily functioning because of physical limitations. For that reason, it is less likely that scores on these subscales could have increased to a higher level. This so-called ceiling effect could have contributed to the lack of intervention effect on RAND-36 subscales regarding physical functioning.

A limitation of this study arises from the representativeness of our study sample. Participation was voluntary, which unintentionally could have led to an overrepresentation of participants who are highly motivated to increase physical activity. Also, selecting participants who were able to use the Internet led to a sample with a relatively high education level. As a consequence, generalizability of the results toward the general elderly population is limited. On the other hand, our study sample contained overweight, inactive older adults with comorbidities—with exclusion of diabetes—which is representative of the general population and consequently leads to increased generalizability. Adults in our study sample are categorized as being inactive, moderately inactive, and moderately active based on the GPPAQ categories and corresponds with less than 3 hours of physical exercise and/or cycling per week. According to the official GPPAQ guidance document, the *active* category is taken as consistent with achieving goals set by the physical activity (PA) guidelines relating to time spent in MVPA or vigorous physical activity (VPA) [9]. It is important to mention that all other categories require a PA intervention, which was the rationale behind our choice for this GPPAQ-guided cutoff point for inclusion in the DirectLife study. The relative short duration of the DirectLife program (ie, 3 months) should be considered while interpreting the results of this study. Although the effects found on quality of life seem promising, these need to be reinforced by results from a longer-term study. In addition, we have not specifically emphasized the unraveling of determinants of quality of life in this study, such as socioeconomic status, family support, computer literacy, type of environment, and housing status. An in-depth and adequately designed determinant study would be useful to unravel the influence of these determinants on physical activity, biomedical outcomes, and quality of life.

An important strength is that we used validated instruments to measure physical activity and quality of life. However, although accelerometry is one of the state-of-the-art objective measurements of physical activity, two comments are noteworthy. First, our choices for wearing the accelerometers on the right side, as well for considering an assessment day as valid if >10 hours were registered, were arbitrary. A study by Masse and colleagues critically used the criteria of different algorithms to reduce accelerometer data on physical activity and showed that the algorithm we used—minimal amount needed: 5 days; minimal daily wear: 10 hours—will not lead to different MVPA scores in comparison to more or less stringent algorithms [22]. Second, the conversion of accelerometer data into an accurate and reliable PA measurement is an evolving topic in PA research. Whether accelerometer data should be used in the evaluation of PA guideline adherence remains under doubt [23]. In our study, still-limited information is available on the type of physical activity that was performed. Knowing whether reduced sitting time or increased cycling or walking time are responsible for improvements of metabolic outcomes and quality of life could contribute to more individually tailored advice on how to improve health and quality of life in the elderly. For example, ankle-worn accelerometers might yield data that is more predictive for other types of physical activity, such as cycling. Activity counts assessed with use of ankle-worn accelerometers are available from a subset of our study population. This topic has our attention and is one of the planned further investigations with data from the study.

Our findings show the feasibility and potential of Internet-assisted physical activity interventions in an older adult population. Based on our findings, we encourage further implementation of the DirectLife program. The DirectLife program 2.0 could function as an open-access, Web-based lifestyle advice tool that can be referred to by general practitioners and medical specialists, or can be consulted by inactive older adults. Further development of the program, however, requires reinforcement of the found effects at a longer term and optimization of the personally tailored advice by including more determinants of physical (in)activity, such as sitting time.

### Conclusions

Our study shows that an Internet-based physical activity program was effective in improving health-related quality of life among inactive 60-70-year-olds that successfully reached their individually targeted increase in daily physical activity after 3 months, as indicated by the intervention program. Our results indicate that significant improvements in physical activity can lead to an improvement of mental functioning in particular.

## Appendix

Multimedia Appendix 1 CONSORT-EHEALTH Checklist v.1.6.1.

## Abbreviations

BMI: body mass index
EMGO+: ExtraMuraal Gezondheids Onderzoek
GPPAQ: General Practice Physical Activity Questionnaire
HbA1C: glycated hemoglobin
HDL: high-density lipoprotein
IQR: interquartile range
LDL: low-density lipoprotein
MVPA: moderate-to-vigorous physical activity
N/A: not applicable
NGI/NWO: Netherlands Genomics Initiative/Netherlands Organisation for Scientific Research
PA: physical activity
QOL: quality of life
RAND: Research ANd Development
RAND-36: Research ANd Development 36-item health survey
VPA: vigorous physical activity
VU: Vrije Universiteit
